# Testing the Role of Climate Change in Species Decline: Is the Eastern Quoll a Victim of a Change in the Weather?

**DOI:** 10.1371/journal.pone.0129420

**Published:** 2015-06-24

**Authors:** Bronwyn A. Fancourt, Brooke L. Bateman, Jeremy VanDerWal, Stewart C. Nicol, Clare E. Hawkins, Menna E. Jones, Christopher N. Johnson

**Affiliations:** 1 School of Biological Sciences, University of Tasmania, Hobart, Tasmania, Australia; 2 SILVIS Lab, Department of Forest & Wildlife Ecology, University of Wisconsin-Madison, Madison, Wisconsin, United States of America; 3 Centre for Tropical Biodiversity and Climate Change and eResearch Centre, James Cook University, Townsville, Queensland, Australia; University of Queensland, AUSTRALIA

## Abstract

To conserve a declining species we first need to diagnose the causes of decline. This is one of the most challenging tasks faced by conservation practitioners. In this study, we used temporally explicit species distribution models (SDMs) to test whether shifting weather can explain the recent decline of a marsupial carnivore, the eastern quoll (*Dasyurus viverrinus*). We developed an SDM using weather variables matched to occurrence records of the eastern quoll over the last 60 years, and used the model to reconstruct variation through time in the distribution of climatically suitable range for the species. The weather model produced a meaningful prediction of the known distribution of the species. Abundance of quolls, indexed by transect counts, was positively related to the modelled area of suitable habitat between 1990 and 2004. In particular, a sharp decline in abundance from 2001 to 2003 coincided with a sustained period of unsuitable weather over much of the species’ distribution. Since 2004, abundance has not recovered despite a return to suitable weather conditions, and abundance and area of suitable habitat have been uncorrelated. We suggest that fluctuations in weather account for the species’ recent decline, but other unrelated factors have suppressed recovery.

## Introduction

Detecting, diagnosing and halting species declines are some of the most challenging tasks faced by conservation practitioners [1]. Timely action is critical to species recovery [2]. Therefore conservation managers are often forced to act on incomplete knowledge of key threats and factors causing the decline [3]. However, unfounded assumptions as to the causal factors can lead to inaccurate predictions of extinction risk and wasted management effort [4, 5].

The eastern quoll (*Dasyurus viverrinus*) is a medium-sized marsupial carnivore that was once widespread in south-eastern Australia. The last confirmed sighting on the Australian mainland was in Sydney in 1963 and the species survives only on the large island (68 400 km^2^) of Tasmania, including Bruny Island [6]. In Tasmania, findings from a large-scale monitoring program using transect counts suggest there was a marked decline in abundance of the species in the early 2000s; this evidence is supported by longitudinal sampling of populations by live-trapping [7]. As a result, the species was listed as Endangered under IUCN criteria in the recent Action Plan for Australian Mammals [8]. The reasons for this decline are unknown. Population irruptions and declines have been anecdotally reported for the species over more than a century [9], suggesting that marked fluctuations may simply be part of the species’ natural history. Similar eruptions in other species have been attributed to short-term changes in rainfall and temperature [10, 11]. If eastern quolls are also sensitive to short-term variations in weather, it is possible that the recent quoll decline may have been driven by a period of unsuitable weather, and that abundance can be expected to recover when optimal conditions return.

Climate exerts a strong influence on the distribution and abundance of many species [12, 13]. Unfavourable climatic conditions may exceed a species’ physiological tolerances [14], limit food resources [15] or disrupt reproduction and completion of life cycles [16]. Long-term changes in climatic conditions can gradually erode environmental suitability, leading to asynchronous feeding and breeding cycles [17] and shifts or reductions in distribution and abundance [18, 19]. Over shorter time scales, fluctuations in weather and extreme events can cause sudden changes in distribution and abundance [20]. For some species, the decline in abundance may be temporary and recovery will ensue without management intervention, while for others it may contribute to a cumulative or permanent trajectory towards extinction [15, 21]. Many declines due to climate change will probably be stepwise rather than smooth, as the changing climate ushers in extreme weather events that cause abrupt declines. One challenge for conservation managers is to quantify the effects of these short- and long-term climatic changes and distinguish them from other possible threatening processes.

Correlative species distribution models (SDMs) use suites of environmental variables to explain observed patterns of species occurrence [22–24]. Such models are based on the premise that a species' current distribution is a good indicator of the environmental requirements for its persistence [25]. Climatic SDMs typically use long-term climatic means to define the climatic niche, thereby producing static depictions of distribution that are assumed to be in equilibrium with the current climate [26]. However, by using temporally explicit occurrence and climatic data, weather SDMs provide additional information on changes in the amount and distribution of climatically-suitable space over time [27, 28]. Such changes are not captured by models using long-term climate means which may not represent the conditions experienced by individuals of short-lived species throughout their lifetime [29]. As the relationship between abundance and environmental suitability is generally positive [30, 31], SDMs that predict temporal variation in the area of suitable habitat for a species may also predict changes in abundance.

In this study, we tested the hypothesis that the recent decline of the eastern quoll in Tasmania is due to short-term variation in climatic variables. We built SDMs for the species using both long-term climate means and short-term weather variables, and we compared the predictions of the area of suitable habitat from the weather model with an index of range-wide abundance of the quoll from standardised transect counts. We made four predictions: (1) climatic variables would provide meaningful predictions of habitat suitability for the eastern quoll; (2) weather SDMs using short-term spatially and temporally explicit weather data would perform better than climate SDMs that use long-term climatic means; (3) weather SDMs would predict a reduction in the amount of suitable habitat corresponding to the period of decline in quoll abundance, and quoll declines would be greatest in regions with lowest mean habitat suitability; and (4) predicted habitat suitability would exhibit a positive relationship with quoll abundance.

## Materials and Methods

### Study species

The eastern quoll is widespread in Tasmania but occurs primarily across the drier eastern half of the island [32]. It is commonly associated with forest-pasture interfaces that provide open grassland for foraging and adjoining natural forest habitat for denning [33], but also occurs in sub-alpine buttongrass (*Gymnoschoerus sphaerocephalus*) moorlands, sedgelands and a mix of wet and dry sclerophyll forest; however it is absent from large tracts of rainforest [7, 34, 35]. It is predominantly insectivorous, although small mammals, birds, reptiles, blackberries (*Rubus fruticosus*) and other plant matter are also eaten, depending on location and seasonal fluctuations in local prey availability [33, 36, 37].

### Species distribution modelling

We collated 1590 eastern quoll occurrence records from the Tasmanian Natural Values Atlas database [38]. Records were spread across the time period from 1955 to 2009 and included museum specimens, incidental observations and a range of standardised trapping, spotlighting and camera trap surveys. Observations with date accuracy > 1 month or location accuracy >10 km were excluded. This ensured that the spatial accuracy threshold for occurrence records was no more than double the resolution of the climatic and weather data (~5 km), thereby reducing the likelihood of covariate errors arising from coarse-resolution observations [39]. To minimise spatial bias from localised survey effort, multiple records within a 5 km radius in the same month and year were treated as a single occurrence record.

Monthly climatic data were obtained at a 0.05° grid scale (~5 km x 5 km) for the period 1947 to 2012 from the Australian Water Availability Project (AWAP) [40]. The spatial resolution of these data was approximately double the maximum home range size for the eastern quoll [33] and therefore was considered appropriate for this species.

We selected eight climatic variables judged to be relevant to the species’ ecology while minimising highly inter-correlated variables. As the species is commonly found in the drier eastern half of Tasmania, we incorporated four precipitation variables derived from the monthly AWAP data (annual precipitation, precipitation of wettest quarter, precipitation of driest quarter and precipitation seasonality measured as coefficient of variation). As insects are a major dietary item for quolls and are affected by environmental temperatures [41], we also included four temperature variables (mean annual temperature, maximum temperature of warmest month, minimum temperature of coldest month and temperature seasonality (coefficient of variation)). Long-term climate means for each of the eight variables were calculated for the 30-year period from 1976 to 2005. Around 75% of the quoll occurrence records were contained within this period, thereby ensuring that the recommended 30-year climate baseline closely matched the temporal spread of presence records used to build models [42]. Short-term weather variables were calculated for the 12-month and 36-month periods immediately preceding each month, from January 1950 to December 2009. Because the eastern quoll is an annual breeder with a short, synchronised mating season [43], the use of variables calculated for periods less than 12 months was not considered appropriate, as an increase in abundance in response to favourable climatic conditions can occur only once a year. The inclusion of 36-month variables allowed for possible cumulative or lag effects on survival or reproductive success in response to environmental conditions accruing throughout the quoll's 3 to 4 year lifetime [33].

We developed SDMs using the algorithm Maxent (version 3.3.3) [24]. Maxent uses presence-only records to relate environmental variables to species occurrences on the basis of maximum entropy [24]. All default settings were used except threshold and hinge features, as this produces more ecologically realistic response curves and provides more general predictions [44]. Climate models were built by relating the 30-year climate means for each of the eight environmental variables to the occurrence records. Weather models were built by relating both the 12-month and 36-month temporally explicit data for each of the eight environmental variables to the month-year and location of each quoll record. To minimise the risk of over-fitting, we reduced the number of highly inter-correlated variables by including only one of the 12- or 36-month versions of each variable in the final model (see S1 Table). These were selected based on their respective permutation importance, which indicates the dependence of the model on that variable, normalised to percentages [45]. For the final weather model, the 12-month data were selected for annual mean temperature and the 36-month data were used for the remaining seven variables. All pairwise Pearson correlations between retained variables in the final model were < ±0.85 [23, 46] as the Maxent algorithm can handle such correlations [23, 46, 47].

We also converted the default Maxent logistic probability distribution from the final weather model to a binary prediction of suitable/unsuitable habitat using a threshold based on equalising training sensitivity and specificity [48, 49]. This threshold provided a strict level of discrimination, thereby predicting those areas most likely to represent core habitat for the species, while still predicting a realistic depiction of its known distribution [50].

The final weather model was projected onto spatial surfaces consisting of the variables across Tasmania for each calendar month from January 1950 to December 2009, thereby producing a single spatially explicit projection for each month for each of the logistic and binary outputs. For each grid cell, the 720 individual monthly values for environmental suitability from 1950 to 2009 were averaged to give a mean value of suitability for that grid cell on a scale of 0 to 1. These average values for each grid cell were used to create a static map depicting the mean geographic distribution of weather-defined suitable habitat for the species.

To account for spatial bias in occurrence records [51, 52], we replaced the uniform background data with a ‘target-group’ background created using occurrence records of related marsupial carnivore species. These species would be expected to be captured or observed using the same survey methods as the eastern quoll, and would therefore be drawn from the same sampling distribution [53]. In this way, the background sample reflected the same bias as our presence data, factoring out any sample-selection bias [54]. The target-group comprised the 1590 eastern quoll records and an additional 6655 occurrence records for the spotted-tailed quoll (*Dasyurus maculatus*) and the Tasmanian devil (*Sarcophilus harrisii*) sourced as for the eastern quoll records for the time period 1955 to 2009. The total 8245 records were scaled up to create a target-group background consisting of 100 000 random points weighted in direct proportion to both the temporal and spatial distribution of the carnivore occurrence records. The spatio-temporal biases were maintained by drawing from the unique spatial locations with a frequency represented by the empirically unique month-year combination observed. This target-group background was used in all climate and weather models.

We used 10-fold cross-validation to assess model fit [26]. This allowed variance estimates to be calculated and evaluated relative to the mean results of the 10 replicate runs. Model performance was evaluated using the area under the receiver operating curve (AUC) [23, 24]. The AUC ranges from 0 to 1, where 1 indicates perfect discriminatory ability, 0.5 indicates no better than random and > 0.75 can provide useful discrimination [23]. With presence-only data, the maximum AUC will be < 1 and is smaller for wide-ranging species [55, 56].

### Relationship between habitat suitability and abundance

The total annual quoll sightings recorded in the Tasmanian state government’s annual vehicle-based spotlight surveys [38, 57] were used as an abundance index (AI) from 1990 to 2009. Each survey was driven along a 10 km transect at a constant speed of 25 km/hr. Survey protocols were standardised where possible for variables such as observer height from ground, type of spotlight, vehicle survey speed, rain, fog and moon phase to help preserve consistency of data, ensure repeatability, reduce observer bias and increase precision and validity of observations [58]. While almost 200 transects are currently surveyed between November and February each year, not all transects have been surveyed in all years. Transects are categorised into 30 regions, each containing 3–8 transects grouped by geographic proximity. Due to the extensive spatial coverage across Tasmania, each transect is surveyed only once each year. The lack of replication within each year, together with variability inherent in this type of survey technique, means that the use of this data is restricted to presence only applications or to long-term trends in abundance across broad spatial scales. The precision and accuracy is not considered sufficient for assessing short-term changes in abundance at regional or transect scales. While these surveys were originally designed to monitor species subject to harvesting (common brushtail possum *Trichosurus vulpecula*, Tasmanian pademelon *Thylogale billardierii* and Bennett’s wallaby *Macropus rufogriseus*), they were found to be useful for monitoring long-term statewide trends in other less frequently detected species, including the eastern quoll [59], and have been corroborated with trends from trapping surveys for the period 1990 to 2009 [7]. Accordingly, these surveys were used for the eastern quoll AI as they provided the broadest spatial coverage of the island, used standardised protocols across years, and were performed around the same time of year annually, thereby reducing the impact of any seasonal effects.

To investigate the spatial relationship between habitat suitability and eastern quoll abundance, the 10-year change in mean AI for each region was overlaid onto the binary core habitat SDM to visually explore whether the largest declines occurred in areas of lowest habitat suitability. For each transect, we compared the mean annual quoll sightings from 1997–99 with those from 2007–09. A 3-year mean was used to reduce the impact of interannual variation in factors that may affect detection probability between years, such as change in observer or differences in time of year or night. The mean annual sightings were then totalled for each region to quantify regional changes in quoll AI over the 10 years to 2009. Regional changes in sightings were previously quantified over this 10 year period in accordance with defined criteria for assessing threatened species status at state, federal and international levels [7]. Only the 150 transects consistently surveyed every year during these two periods (1997–99 and 2007–09) were included in the AI for this analysis. Eight regions were excluded from this analysis, as there were either no eastern quoll detections in the region during the 10 year period (7 regions), or there was no change in the AI over the 10 year period (1 region). As the data precision was not considered adequate for robust quantitative analyses at the regional or transect scale, our assessment was performed using a visual exploratory analysis only.

To investigate the temporal relationship between habitat suitability and quoll abundance, we also compared the total quoll AI to the total area of core habitat across Tasmania each year between 1990 and 2009. While 150 transects were used in the regional analysis for the 10 years to 2009, not all of these transects were surveyed every year between 1990 and 2009. Accordingly, for this long-term analysis, we omitted the 3 transects with incomplete data and only included the 147 transects that were surveyed every year during this 20 year period in the quoll AI. As sightings from spotlight surveys were included in the occurrence records used to build all climate and weather models, we derived the amount of environmentally suitable area from a second independent core habitat SDM. The independent SDM was built as outlined for the previous weather model, however all spotlight survey records were excluded from both the quoll occurrence file and the marsupial carnivore target-group background file used to build the model. In this way, the amount of environmentally suitable habitat derived from this second weather model was independent of the spotlight data used in calculating the AI. The reduced occurrence file excluding spotlight sightings contained 880 eastern quoll records between 1955 and 2009, while the target background file contained a total of 1924 records between 1955 and 2009 for the three carnivore species. Output from this independent weather model was compared to the original weather model to ensure AUC, important variables and geographic distribution did not differ markedly between models. Quoll AI was compared graphically with the total area of suitable habitat from the independent binary output from the weather model from 1990 to 2009. Changepoint analysis was performed using the changepoint package version 1.1.5 [60] to identify two key changepoints: (1) the year when mean quoll AI changed, and (2) the year when the relationship between the amount of suitable habitat and quoll AI changed (as defined by the ratio of total suitable area:quoll AI). Changepoint analysis uses a maximum log-likelihood approach to determine the point in a time series where the mean or the variance changes [61]. For each analysis, we tested for a single changepoint and assumed that the data was distribution-free [62]. The quoll AI was log-transformed to stabilise the variance, and linear regression was used to model the amount of suitable area against the log of quoll AI for each year. Separate regressions were performed before and after the second changepoint and were compared to investigate how the relationship between suitable habitat and quoll AI changed.

All statistical analyses were performed using R version 3.0.1 [63].

## Results

### Distribution models

**Fig 1:**
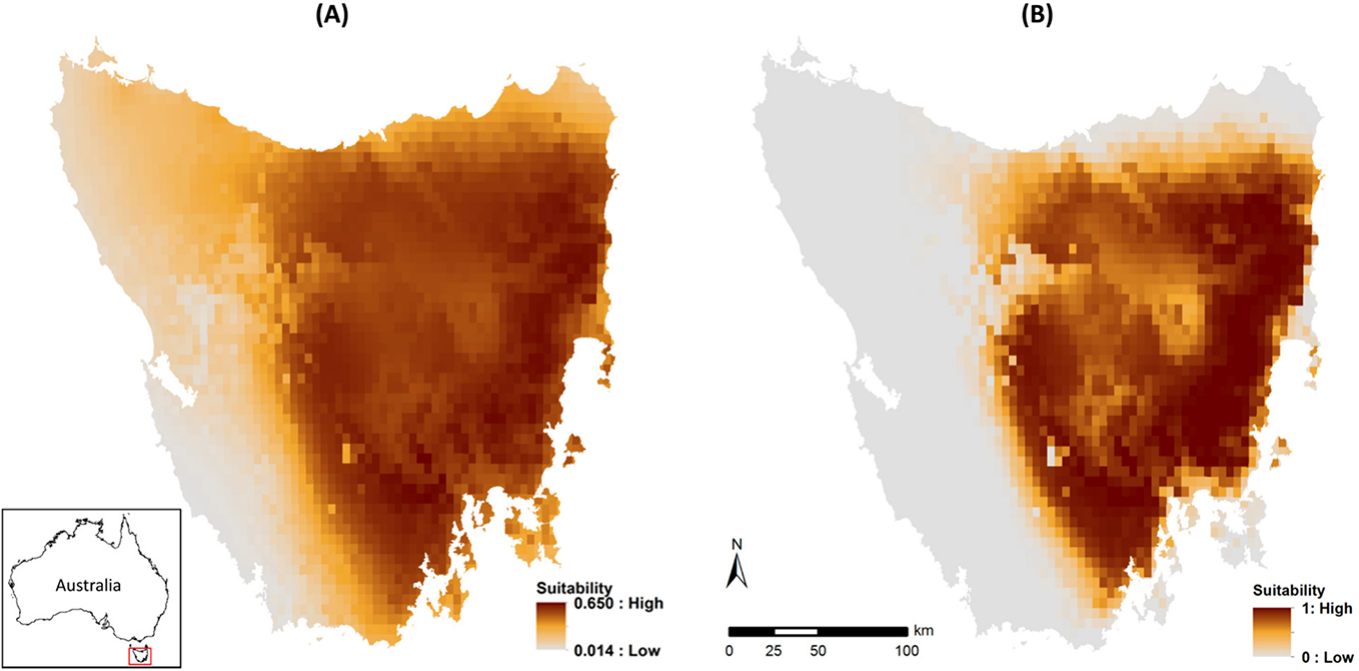
Weather-defined species distribution models for the eastern quoll in Tasmania, showing (A) habitat suitability (logistic output) and (B) core distribution (binary output). Projections are a composite of the 720 individual monthly projections between January 1950 and December 2009 (individual monthly projections shown in S1 Video). The value for each 5 km x 5 km grid cell represents the average suitability for that cell across the 720 individual months. Grey shading indicates not suitable, with increasing suitability shown from orange to red. Inset shows location of Tasmania within Australia.

Both the climate model (mean AUC ± s.d. = 0.774 ± 0.011; S1 Fig) and the weather model (AUC = 0.755 ± 0.019; Fig 1) provided meaningful predictions of habitat suitability that were consistent with the known distribution of the eastern quoll. While there was no marked difference in model fit, the most important variables differed between models. Precipitation of the driest quarter (37.8%), precipitation seasonality (18.4%) and annual precipitation (15.5%) had the highest permutation importance for the climate model, while precipitation of wettest quarter (38.6%) and minimum temperature of the coldest month (37.0%) were the most important variables for the weather model (S2 Fig). Environmental suitability was negatively associated with all precipitation variables in all models, with highest predicted suitability in areas of low or no precipitation (S2 Fig). The minimum temperature of the coldest month was positively related to quoll occurrence at temperatures below 0°C, but negatively related at temperatures above 0°C (S2 Fig).

Performance of the second independent weather model (mean AUC = 0.738 ± 0.014) was consistent with the full weather model. The most important variables and their relationship with likelihood of quoll occurrence did not differ between weather models, with minimum temperature of the coldest month (40.7%) and precipitation of wettest quarter (35.9%) having the highest permutation importance in the second model (S2 Fig).

### Relationship between habitat suitability and abundance

**Fig 2:**
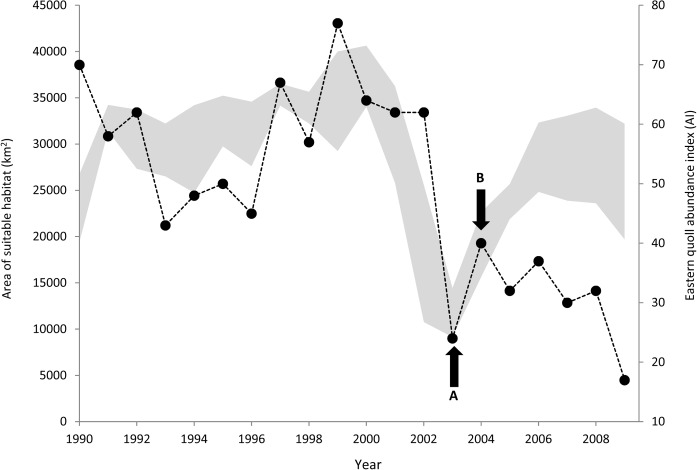
Temporal variation in area of environmentally suitable habitat and quoll abundance from 1990 to 2009. Grey shading represents the total area of core habitat across all 12 months for each year (left axis) as given by the independent binary weather model. Width of shading indicates variability of suitable area within each year (lower and upper bounds of shading represent the months with the lowest and highest amounts of suitable habitat respectively). Black dots represent the quoll abundance index (AI), being the total number of eastern quoll sightings recorded in annual spotlight surveys across all transects (*n* = 147) surveyed every year from 1990 to 2009 inclusive (right axis) [7]. Arrows indicate (A) identified changepoint in mean quoll AI, and (B) identified changepoint in relationship between area of suitable habitat and quoll AI.

The total area of core habitat fluctuated considerably through time (mean: 29 054 km^2^, range: 7200–49 625 km^2^) (S1 Video). A changepoint in mean quoll AI was identified in 2003, reducing from 56.357 ± 3.591 sightings between 1990 and 2003 down to 31.333 ± 3.242 sightings thereafter. The relationship between suitable area and quoll AI changed one year later in 2004. Temporal trends in the quoll AI were positively correlated with the total amount of core habitat each year between 1990 and 2004 (*r* ^2^ = 0.269; *F*_*1*,*13*_ = 4.790; *P* = 0.047), including a marked decline in both suitable area and AI between 2001 and 2003 (Fig 2) when winter minimum temperatures were warmer and precipitation in the wettest quarter was higher. After 2004, quoll AI remained low despite a steady increase in the amount of suitable habitat between 2005 and 2009 (*r* ^2^ = 0.010; *F*_*1*,*3*_ = 0.030; *P* = 0.873) (Fig 2).

**Fig 3:**
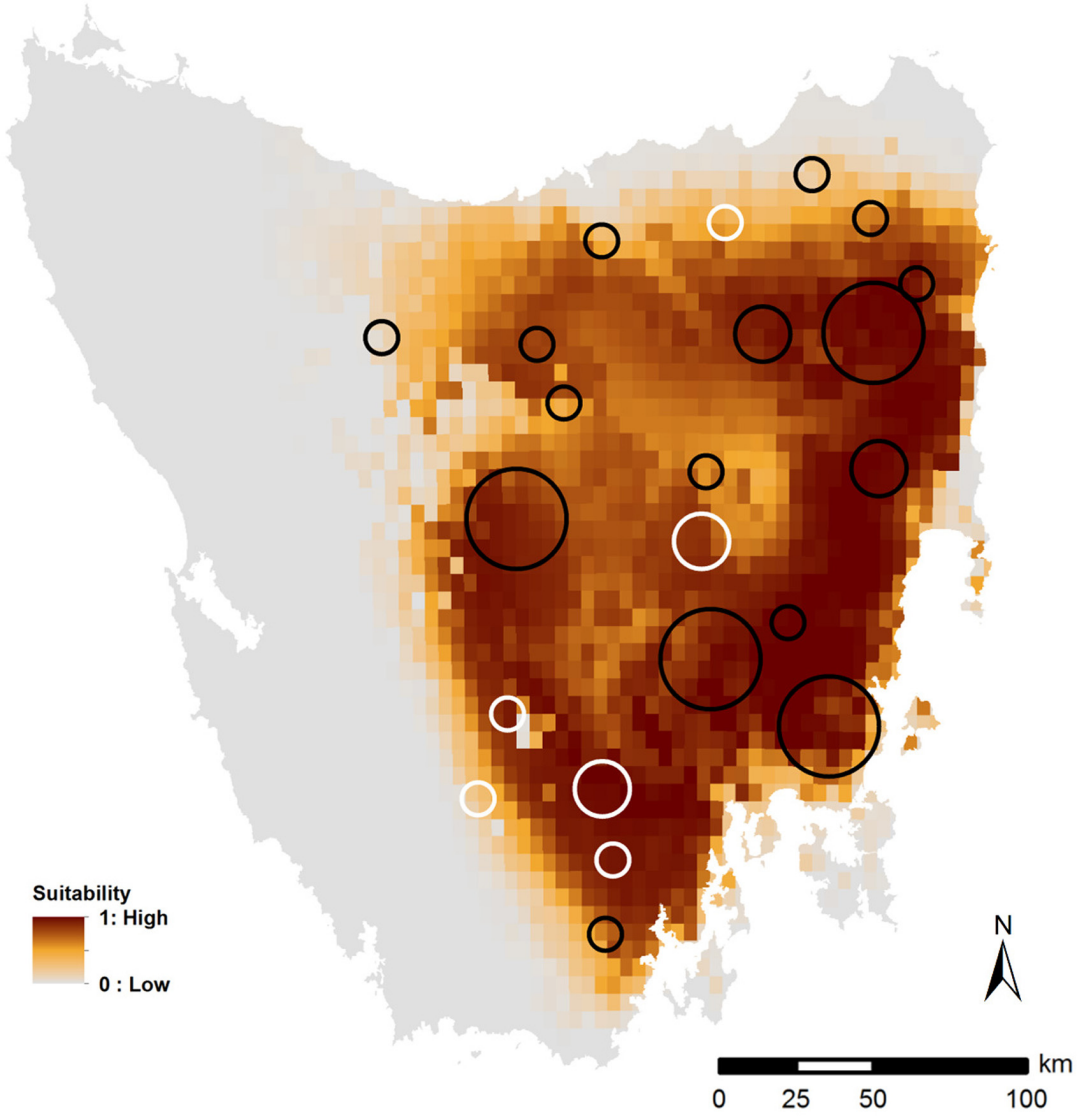
Map of Tasmania showing spatial distribution of changes in mean index of eastern quoll abundance (AI) by region over the 10 years to 2009, as recorded in annual spotlight surveys, overlaid onto the predicted core habitat distribution for the eastern quoll as defined by the binary weather model. Change in AI is calculated as the change in the mean annual number of quoll sightings from 1997–99 to 2007–09 for each spotlighting region, based on the 150 transects consistently surveyed in each of these two periods [7]. White circles indicate an increase in AI, black circles indicate a decrease in AI for each region. Circle size indicates relative magnitude of absolute increase or decrease in AI, being large circles (>6 quoll sightings), medium circles (3–6 sightings), and small circles (<3 sightings). Eight regions have been excluded from this analysis, as there were either no eastern quoll detections in the region during the 10 year period (7 regions), or there was no change in the AI over the 10 year period (1 region).

Spatial patterns of decline in the AI did not match our predictions. The four regions that sustained the greatest decline in abundance in the 10 years to 2009 (76% of island-wide decline in AI over that period) were all located within core areas supporting the highest levels of habitat suitability and stability (Fig 3). Regions that experienced the smallest declines in abundance were predominantly located along core habitat margins where habitat suitability was lower.

## Discussion

We used temporally-explicit weather SDMs to show the contribution of short-term variability in climate to the recent decline of a threatened species. As predicted, fluctuations in abundance of the eastern quoll in recent decades, including a sharp decline between 2001 and 2003, were related to changes in weather across the species’ range. More recently this relationship appears to have broken down, however, so that while weather conditions improved after 2004, there has been no corresponding recovery in abundance of eastern quolls. Possibly, the recovery of quolls is now being prevented by some factor unrelated to climate and weather. If so, the recent decline may not be temporary and recovery is unlikely without management intervention.

Both climate and weather models accurately predicted the species' known geographic distribution, suggesting that habitat suitability for the eastern quoll is well characterised by climatic variables. Contrary to our predictions, the discriminative ability and the broader spatial distribution of suitable habitat were similar for both climate and weather models, although differences in suitability were evident at finer spatial scales. This suggests that, when averaged over the 60-year modelling period, weather variables intuitively provide similar predictions of long-term habitat suitability to climate models. However, it is the ability to quantify variation within that 60-year period that demonstrates the value of the weather model as an interpretative tool. While climate models provided information on the long-term suitability of habitat for eastern quolls, the weather model revealed how the distribution of suitable habitat varies through time. This short-term variation in habitat suitability is pertinent to conservation managers trying to understand how short-term variation in weather may affect the distribution and abundance of short-lived species, such as the eastern quoll.

Habitat suitability was highest in areas of low precipitation and where minimum winter temperatures fell to around 0°C. Our predicted distribution of core habitat throughout the drier eastern half of the island is broadly consistent with a previous distribution model [32] and matches the species’ known distribution. However, the mechanisms by which precipitation and temperature influence eastern quolls require further investigation. For example, it is possible that drier areas support larger populations of the insects and rodents that form a substantial part of the eastern quoll’s diet [33, 36]. Minimum winter temperatures may critically influence the species’ highly synchronised breeding, suggested by the observation that mating in high-altitude populations occurs up to two months later in years when winter minimums were delayed and warmer [64]. The model output suggests that the marked decline in predicted area of suitable habitat during 2001 to 2003 could be due to a period of warmer winter temperatures and heavier precipitation. Neither of these predicted shifts was large, but our modelling suggests that in combination, they caused a substantial reduction in climatic suitability for this species. As the frequency of extreme weather events in Tasmania is predicted to increase, specifically warmer temperatures and more intense extreme rainfall events [65], our findings highlight an additional long term management concern for the species.

Intraspecific abundance-distribution relationships tend to be positive, such that species declining in abundance also show declines in distribution, and the converse [31, 66]. Our analysis is consistent with this, in that our predictions of total suitable area for the eastern quoll through time were positively related to an independent measure of variation in relative abundance. Furthermore, the highest quoll abundance (and subsequently the largest 10-year declines in abundance) occurred in regions with the highest predicted suitability, suggesting that suitable weather conditions had facilitated the higher abundance prior to the decline. Conversely, the smallest declines occurred at range margins, where population abundance was lower prior to the decline, consistent with the lower habitat suitability in these regions [67, 68].

The wide disparity between suitable habitat and abundance after 2004 indicates that abundance is now being held below its potential value by some factor not included in our weather model. Detailed monitoring using live trapping, camera surveys and additional spotlight surveys at a number of sites between 2010 and 2013 has revealed continuing population declines, with no signs of recovery [64, 69]. Camera surveys undertaken during 2012–13 confirm that eastern quolls are still widespread (detected at 14 of 17 sites surveyed) across their predicted distribution, although only low numbers of individuals (between 1 and 4 quolls per linear kilometre) were detected at most sites [64, 69]. This suggests that the current low abundance is not due to a contraction in distribution due to local extinctions at range margins, but rather a general reduction of density throughout the range.

While low environmental suitability, as predicted by SDMs such as Maxent, may indicate low abundance [30, 70], abundance may also vary over a wide range in areas of high environmental suitability because other factors can affect whether or not potential abundance is realised. These factors can include habitat type [71], competition [72], predation [73], parasites and pathogens [19], dispersal ability [74] and disturbance [16].

There are a plethora of factors which may be suppressing quoll populations and driving their ongoing decline [7, 64]. Tasmania is currently undergoing a period of ecological upheaval; the red fox (*Vulpes vulpes*) was recently introduced to the island [75, 76], widespread 1080 fox baiting commenced in 2002 [76] presenting a novel threat to eastern quolls [77] and extensive habitat modification and changes in land use have occurred [78]. The severe decline of the island’s largest mammalian carnivore, the Tasmanian devil [79], may be allowing changes in the behaviour of mesopredators such as feral cats (*Felis catus*) that may threaten a range of species, including the eastern quoll [69]. A recent study found that while there was no evidence of an increase in feral cat abundance following devil declines, there was some evidence that cats may be shifting their activity temporally, suggesting that cats may be becoming more nocturnal with increasing time since devil decline [69]. If this is the case, nocturnal eastern quolls may now be facing an increase in predation by feral cats, even without an increase in feral cat abundance.

While threats such as feral cats have been present and likely acting on eastern quoll populations in Tasmania for many decades, historic quoll abundance may have been high enough to sustain the impacts of these and other threats without long-term negative effects on populations. The low quoll abundance observed during 2002–03, however, may have fallen below a critical density threshold from which recovery is difficult or improbable, even in the absence of new threats or increasing severity of existing threats. Small populations are typically more susceptible to extinction through demographic, environmental and genetic stochasticity and natural catastrophes [1, 80, 81]. Once a species is rare throughout much of its geographic range, the loss of even small numbers of individuals can lead to functional extinction and will rapidly result in local population extinctions [82]. In the absence of consistent and reliable abundance records back to 1950, we are unable to determine whether 2002–03 was the first instance between 1950 and 2009 of such low abundance of eastern quolls. However, during this period, the total area of core habitat fell below 15,000 km^2^ in only 34 months, with the 18 months from July 2002 to December 2003 representing the longest consecutive period below 15,000 km^2^. This unprecedented reduction in core habitat and the historic correlation between core habitat suitability and quoll abundance suggests that the low abundance observed during 2002–03 may also have been unprecedented throughout this 60 year period.

## Conclusion

We have demonstrated that the distribution and abundance of the eastern quoll appear to be correlated with changes in short-term weather variables. Temporally explicit SDMs related unfavourable weather conditions to a sudden decline in both distribution of core habitat and quoll abundance. However, while improved weather conditions predicted a subsequent recovery in suitable habitat, quoll abundance did not recover. This suggests that the recent decline in abundance is not a short-term fluctuation, and that some unmeasured factor(s) is continuing to suppress quoll populations and inhibit their recovery. We suggest that while the causal agents continue to operate unchecked, ongoing declines may lead to an increased extinction risk. Further research is required to identify these agents.

## Supporting Information

S1 Fig The predicted range of the eastern quoll in Tasmania as determined by the long-term climate model. Projections are based on 30-year climatic means from 1976–2005 inclusive. Grey shading indicates not suitable, with increasing suitability shown from orange to red. Inset shows location of Tasmania within Australia.
(TIF)

S2 Fig Response curves of eastern quolls for each of the modelled weather variables in Tasmania. **Response curves are shown for (a) full weather model (12 or 36 month variables) and (b) independent weather model (excluding spotlight survey data).** Each curve represents a different Maxent model created using only the corresponding variable. These plots reflect the dependence of predicted habitat suitability both on the selected variable and on dependencies induced by correlations between the selected variable and other variables. For all curves, the y axis indicates how predicted habitat suitability is dependent on precipitation (mm) or temperature (°C) shown on the x axis. Precipitation and temperature seasonality curves reflect the coefficient of variation for each variable. The red curve shows mean response of 10 replicate runs used to cross-validate the model, blue shading indicates ± one standard deviation.
(PDF)

S1 Table Correlation matrices for the eight climatic variables used in weather models for the eastern quoll in Tasmania (1950–2009). (A) The correlations for each of the eight variables between the two time periods (12 months and 36 months). (B) The correlations between the eight variables used in the final weather model.
(PDF)

S1 Video Dynamic weather model, showing monthly variation in eastern quoll core habitat from 1950 to 2012. See attached GIF file.
(GIF)
