# Supplementary figures and images for: Testing the Role of Climate Change in Species Decline: Is the Eastern Quoll a Victim of a Change in the Weather?
Source: PLoS One. 2015 Jun 24;10(6):e0129420. doi: 10.1371/journal.pone.0129420 (PMC4479380; doi:10.1371/journal.pone.0129420)

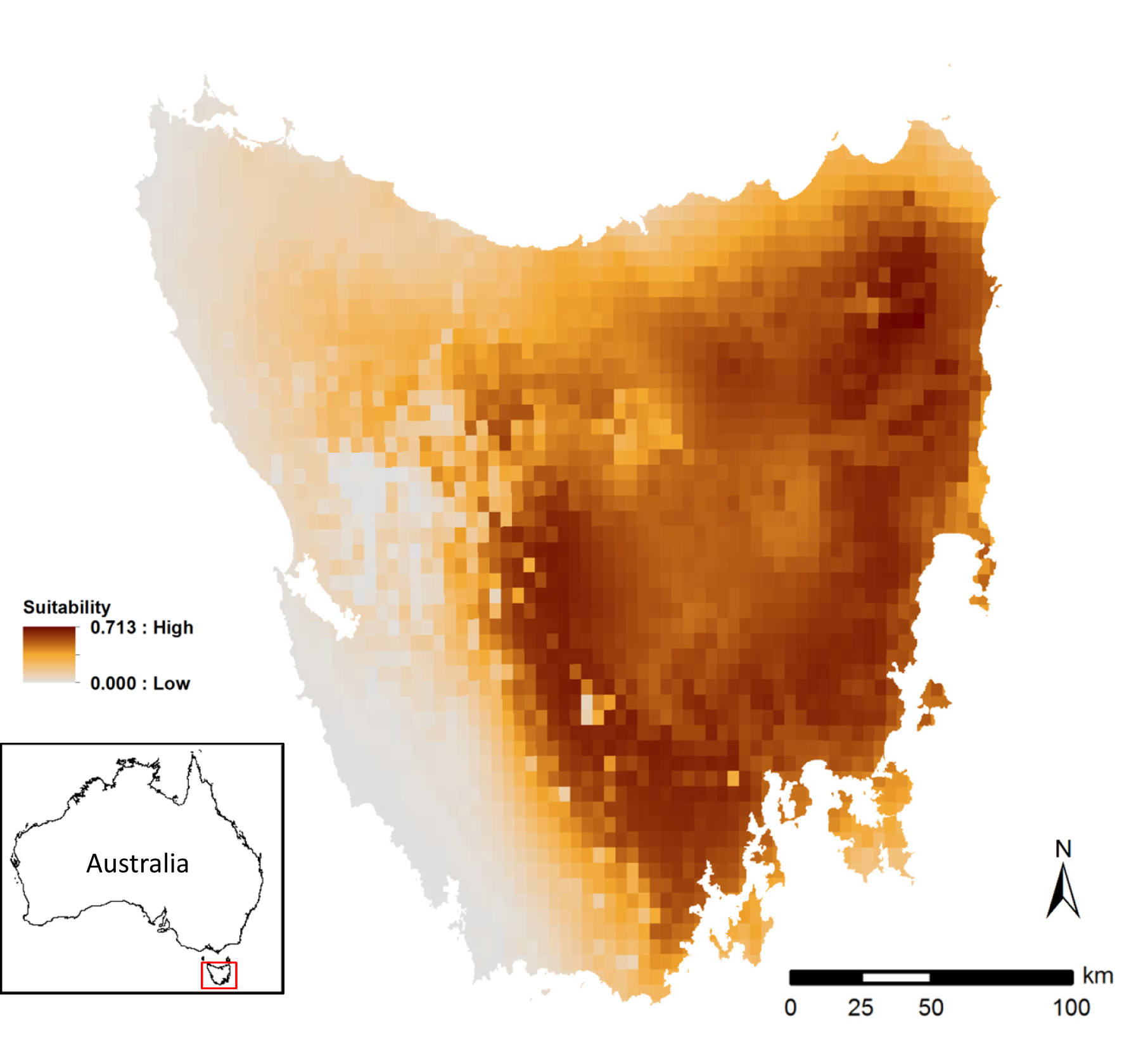

Supplement: S1 Fig — Projections are based on 30-year climatic means from 1976–2005 inclusive. Grey shading indicates not suitable, with increasing suitability shown from orange to red. Inset shows location of Tasmania within Australia. (TIF) [file pone.0129420.s001.tif]

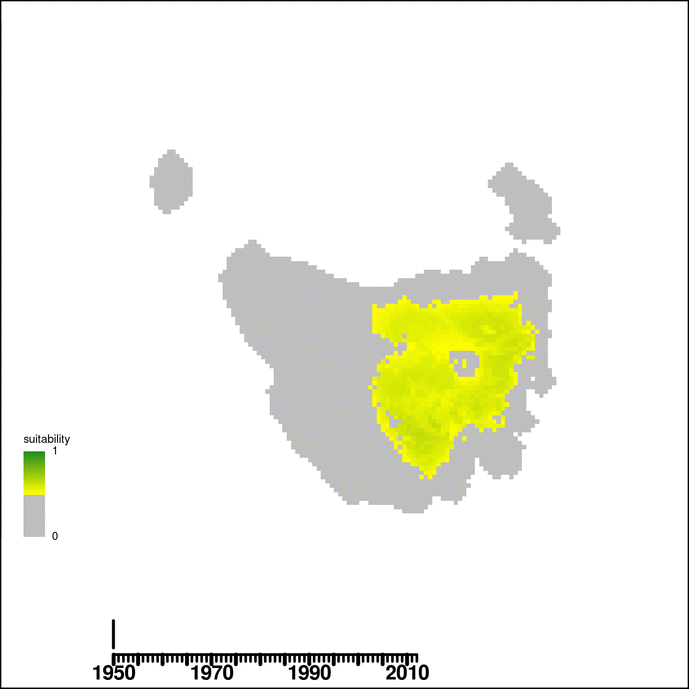

Supplement: S1 Video — See attached GIF file. (GIF) [file pone.0129420.s004.gif]
