# Supplementary material for: Testing the Role of Climate Change in Species Decline: Is the Eastern Quoll a Victim of a Change in the Weather?
Source: PLoS One. 2015 Jun 24;10(6):e0129420. doi: 10.1371/journal.pone.0129420 (PMC4479380; doi:10.1371/journal.pone.0129420)
Supplement: S2 Fig — Response curves are shown for (a) full weather model (12 or 36 month variables) and (b) independent weather model (excluding spotlight survey data). Each curve represents a different Maxent model created using only the corresponding variable. These plots reflect the dependence of predicted habitat suitability both on the selected variable and on dependencies induced by correlations between the selected variable and other variables. For all curves, the y axis indicates how predicted habitat suitability is dependent on precipitation (mm) or temperature (°C) shown on the x axis. Precipitation and temperature seasonality curves reflect the coefficient of variation for each variable. The red curve shows mean response of 10 replicate runs used to cross-validate the model, blue shading indicates ± one standard deviation. (PDF) [file pone.0129420.s002.pdf]

(a) Full weather model

(b) Independent weather model

Annual mean temperature

Temperature seasonality

Maximum temperature of warmest month

Minimum temperature of coldest month

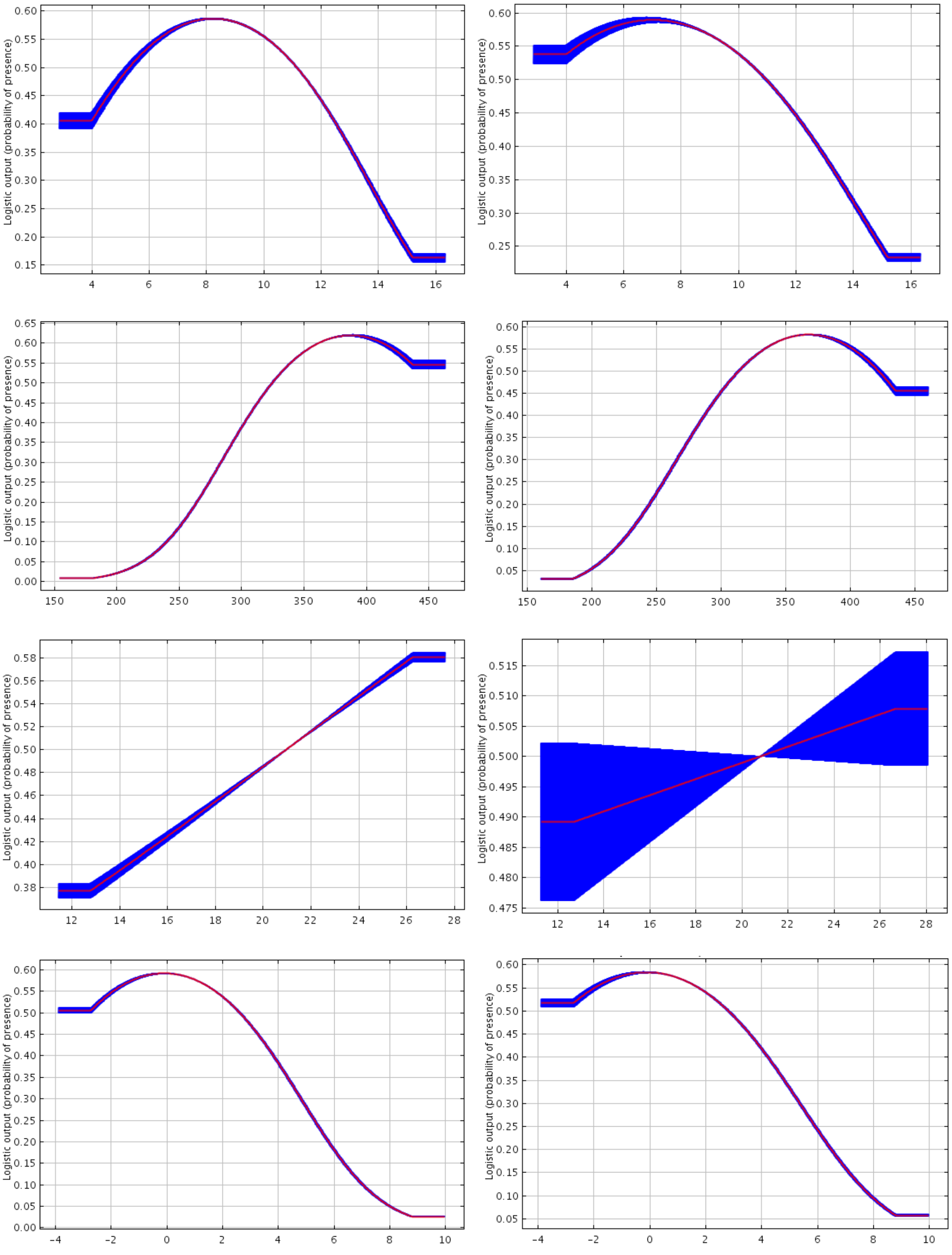

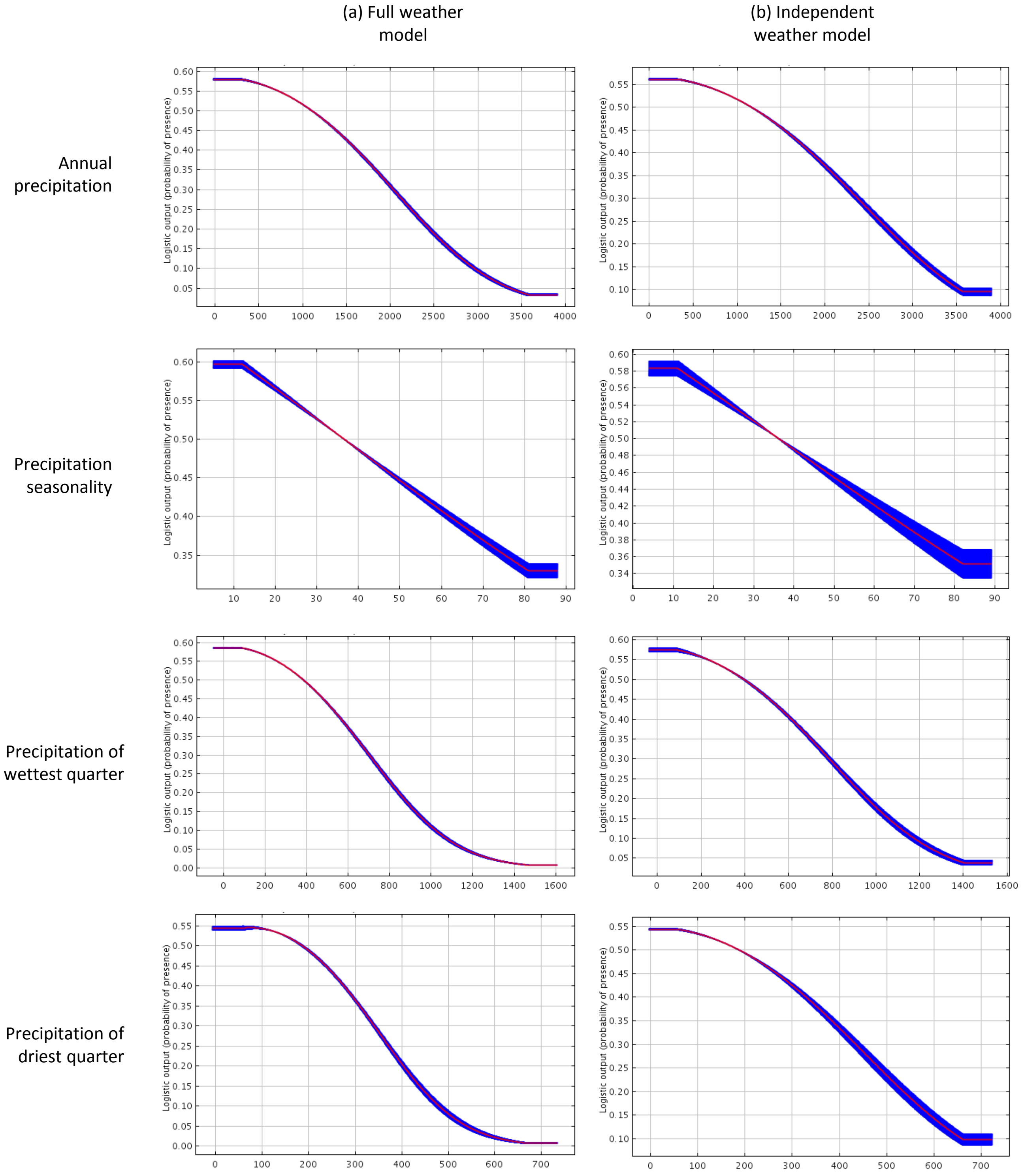

(a) Full weather model
(b) Independent weather model
Annual precipitation
Precipitation seasonality
Precipitation of wettest quarter
Precipitation of driest quarter
Logistic output (probability of presence)
