# Supplementary material for: Testing the Role of Climate Change in Species Decline: Is the Eastern Quoll a Victim of a Change in the Weather?
Source: PLoS One. 2015 Jun 24;10(6):e0129420. doi: 10.1371/journal.pone.0129420 (PMC4479380; doi:10.1371/journal.pone.0129420)
Supplement: S1 Table — (A) The correlations for each of the eight variables between the two time periods (12 months and 36 months). (B) The correlations between the eight variables used in the final weather model. (PDF) [file pone.0129420.s003.pdf]

(A)		36 month
Annual mean temperature (bc01)	12 month	0.80
Temperature seasonality (bc04)	12 month	0.55
Max temperature warmest month (bc05)	12 month	0.56
Min temperature coldest month (bc06)	12 month	0.60
Annual precipitation (bc12)	12 month	0.62
Precipitation seasonality (bc15)	12 month	0.40
Precipitation wettest quarter (bc16)	12 month	0.57
Precipitation driest quarter (bc17)	12 month	0.55

(B)	*bc01.12m*	*bc04.36m*	*bc05.36m*	*bc06.36m*	*bc12.36m*	*bc15.36m*	*bc16.36m*
bc04.36m	0.28						
bc05.36m	0.49	0.70					
bc06.36m	0.47	(0.07)	0.14				
bc12.36m	(0.36)	0.00	(0.24)	(0.07)			
bc15.36m	0.01	0.03	0.25	0.13	(0.04)		
bc16.36m	(0.19)	0.03	(0.13)	0.16	0.82	0.39	
bc17.36m	(0.25)	(0.21)	(0.47)	(0.01)	0.58	(0.61)	0.22
